# Unexplained Perivascular Chorioretinal Atrophy: Is Ranolazine a Possible Cause?

**DOI:** 10.18502/jovr.v21.18696

**Published:** 2026-07-20

**Authors:** Saeed Karimi, Mohsen Zare, Pantea Karbasi, Morteza Hosseini

**Affiliations:** Department of Ophthalmology, Torfeh Medical Center, Shahid Beheshti University of Medical Sciences, Tehran, Iran

**Keywords:** Drug-related Side Effects, Ranolazine, Retinal Toxicity

## Abstract

**Purpose:**

To report a case of unexplained perivascular chorioretinal atrophy temporally associated with ranolazine use.

**Case Report:**

A 64-year-old man with chronic stable angina pectoris was referred to our retina clinic with a complaint of an inferior visual field defect in the right eye over the last 1 month. The symptom onset coincided with the prescription of ranolazine extended-release 500 mg daily and slowly progressed over this period. Central and color vision were normal, and relative afferent pupillary defect was negative. Fundus exam showed chorioretinal atrophy in both eyes along the main vascular arcades, which was more prominent in the right eye and along the superior temporal arcade. Visual field assessment disclosed a double arcuate scotoma in both eyes. Swept-source optical coherence tomography showed outer retina, retinal pigment epithelium, and choriocapillaris atrophy in the extrafoveal area. Fundus autofluorescence showed hypo-autofluorescence, and fluorescein angiography revealed staining along the vascular arcades. Electroretinogram disclosed abnormal photopic and scotopic responses. All findings were more prominent in the right eye and along the superior temporal arcade. Ranolazine intake was stopped. Three months after drug cessation, the patient's visual field and electroretinogram improved.

**Conclusion:**

This is the first case report on ranolazine-associated chorioretinal toxicity, highlighting the necessity of further studies on potential side effects of even short-term exposure to this drug.

##  INTRODUCTION

Ranolazine is a piperazine derivative that inhibits ventricular myocardial sodium and potassium ion channels. It reduces intracellular calcium overload.[1] Ranolazine is FDA-approved for chronic stable angina pectoris (CSAP).[2] It has a half-life of about 7 hours and undergoes hepatic metabolism with cytochrome P450.[3] Reported side effects are dizziness, constipation, and peripheral edema—which are more often seen in patients with hepatic or renal failure.[4]

To the best of our knowledge, there is no report of specific ocular toxicity associated with ranolazine. Here, we present the first report of presumed ranolazine-induced chorioretinal toxicity that developed within 1 month of intake and was confirmed on multimodal imaging, and subsequent clinical and imaging improvement 3 months after drug cessation.

##  CASE PRESENTATION

**Figure 1:**
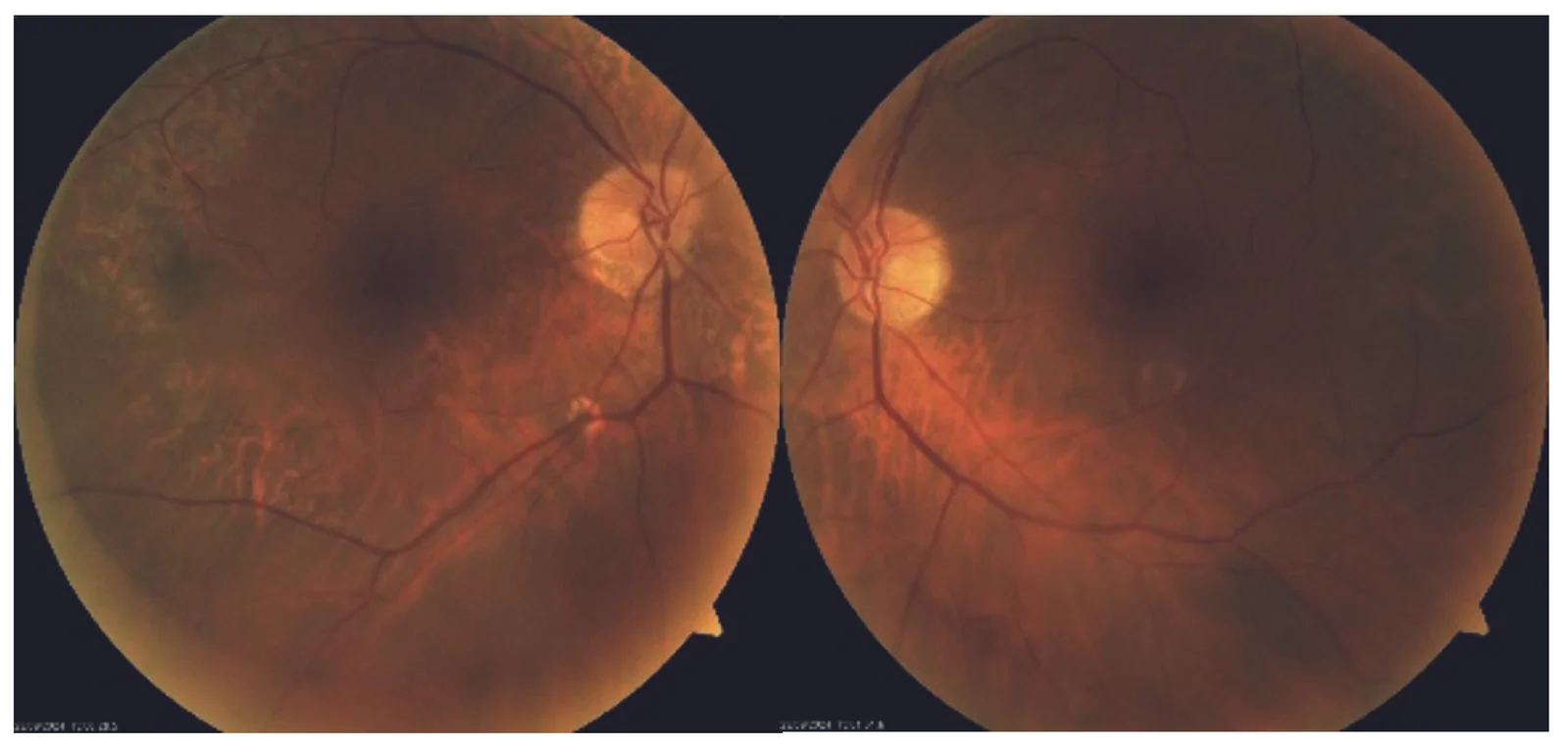
Fundus photograph showing peripapillary atrophy in the right eye and bilateral chorioretinal atrophy along the main superior and inferior vascular arcades. Atrophic changes are more significant along the superior arcades and in the right eye.

**Figure 2:**
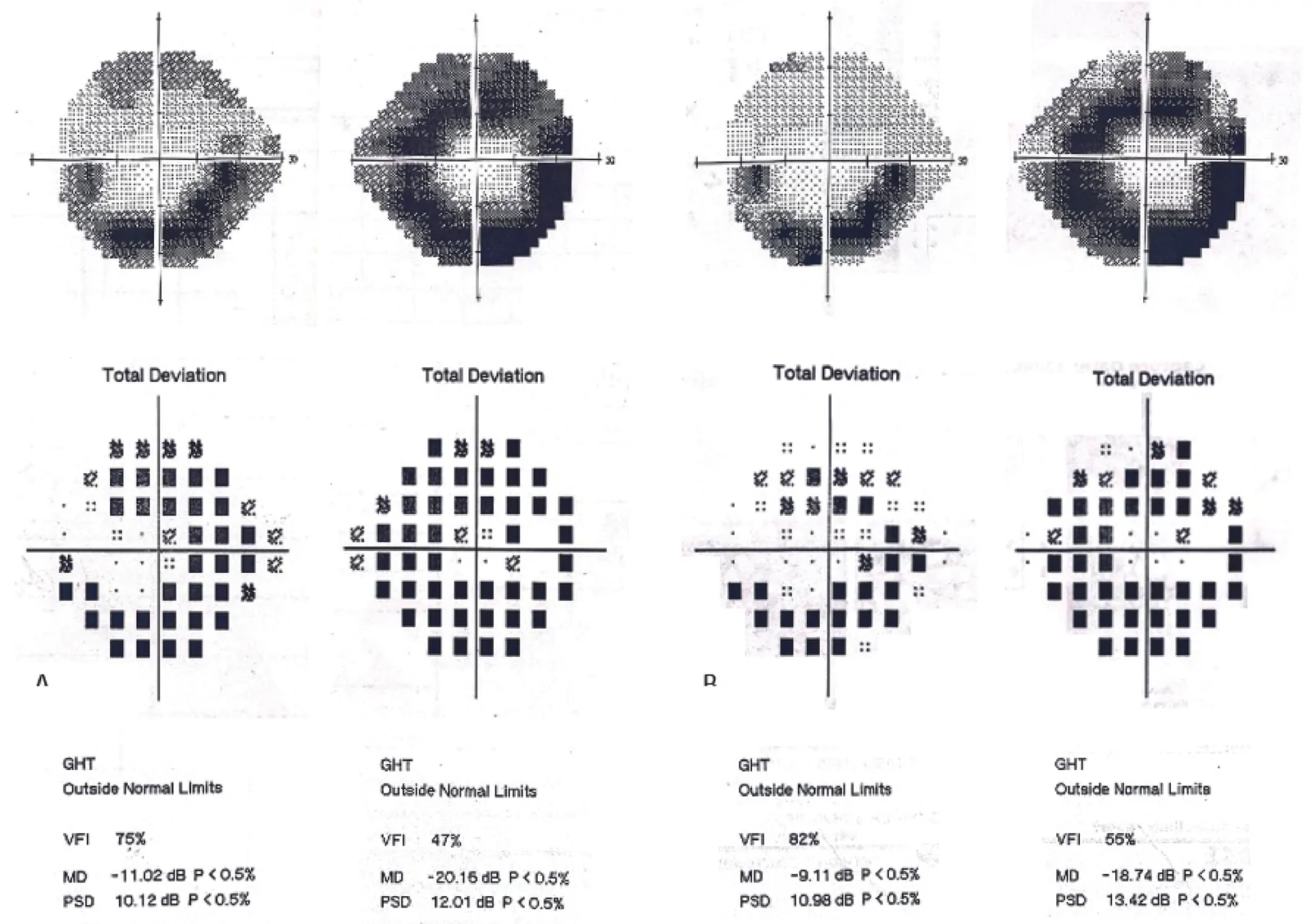
Humphrey Visual Field 24-2 Threshold Test. (A) The initial gray scale and total deviation maps. Both eyes show a double arcuate pattern with central island preservation. Changes in the left eye are milder. Defects are also deeper in the inferior hemifield, which is compatible with more significant atrophy along the superior temporal vascular arcades. A second visual field was obtained in the first exam, with similar results. (B) The same maps 3 months after cessation of ranolazine. Both eyes show improvement. Mean deviation was - 20.16 dB OD and - 11.02 dB OS on initial visual field testing and - 18.74 dB OD and - 9.11 dB OS on follow-up. All tests were reliable, without significant fixation loss, false positive errors, or false negative errors.

**Figure 3:**
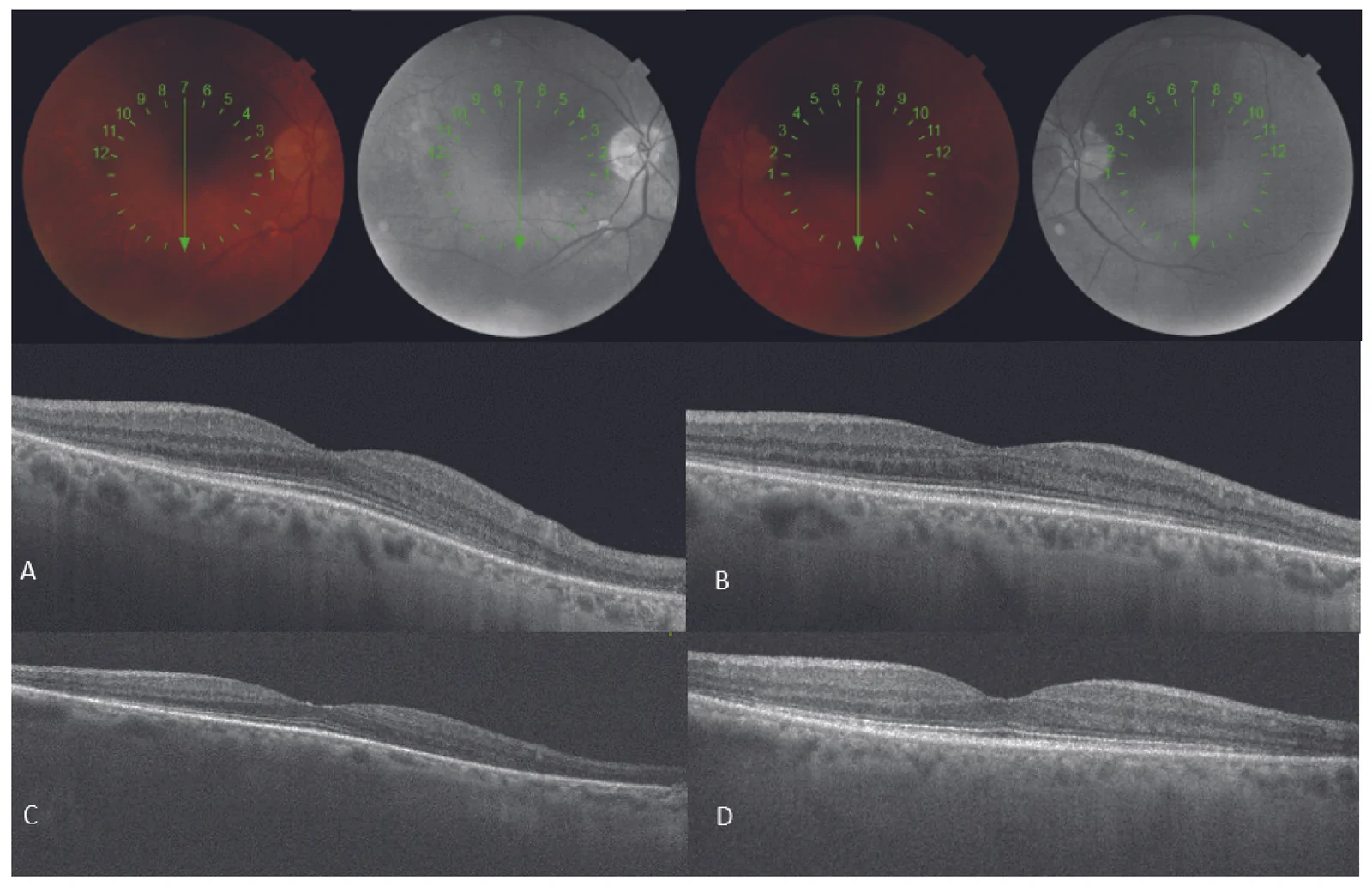
SS-OCT showing ONL, ELM, EL, RPE, and choriocapillaris atrophy in the parafoveal region. (A&B) Initial OCT of the right and left eyes, respectively, using Topcon DRI-OCT Triton SS-OCT. (C&D) OCT of the right and left eyes, respectively, 3 months after ranolazine cessation using Optovue RTVue OCT).

**Figure 4:**
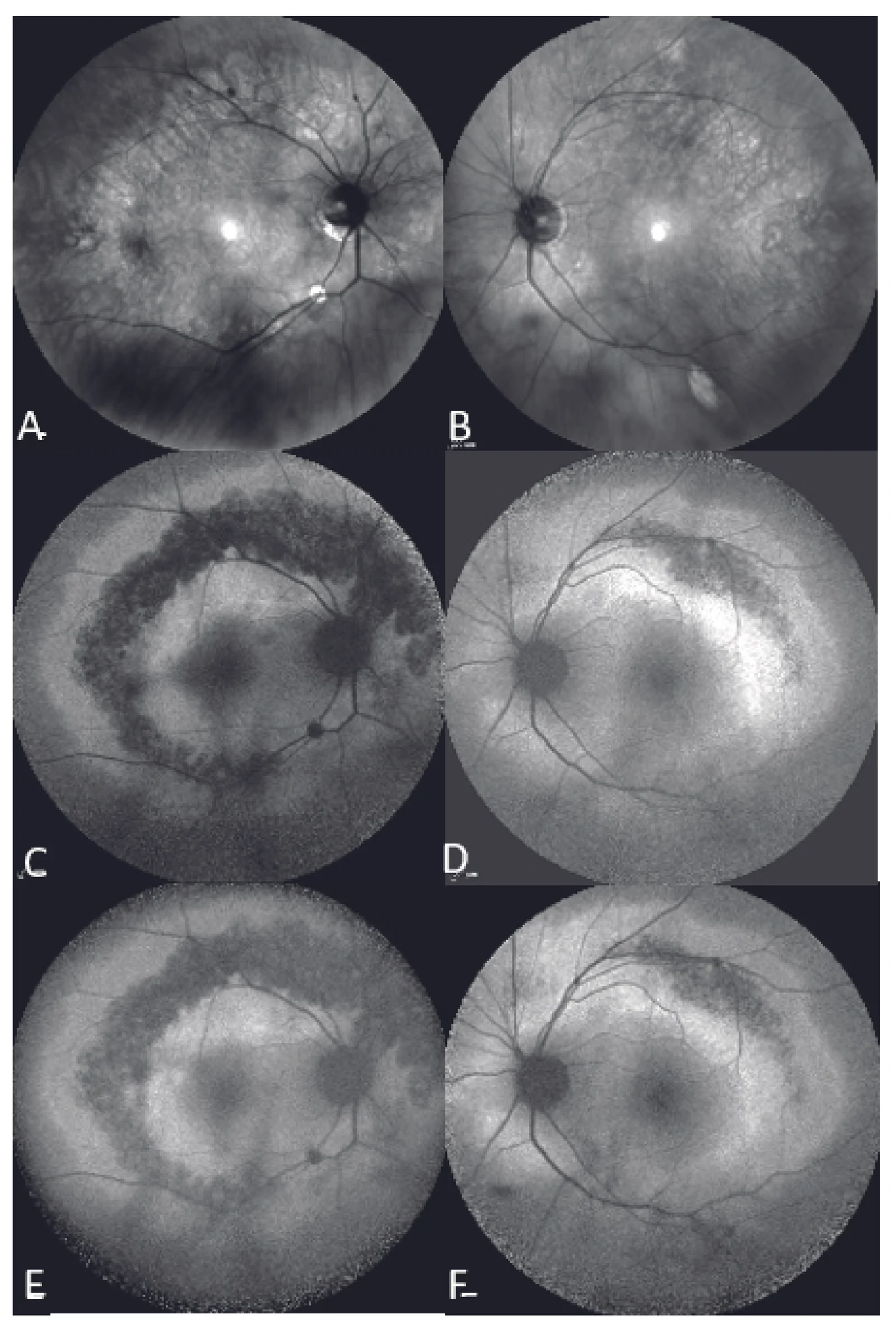
(A&B) Initial infrared image of the right and left eyes, respectively. (C&D) Initial FAF showing hyperautofluorescence in the macular region and hypoautofluorescence along the main vascular arcades in the right eye and along the superior temporal arcade in the left eye. (E&F) FAF remained without significant change after ranolazine cessation.

**Figure 5:**
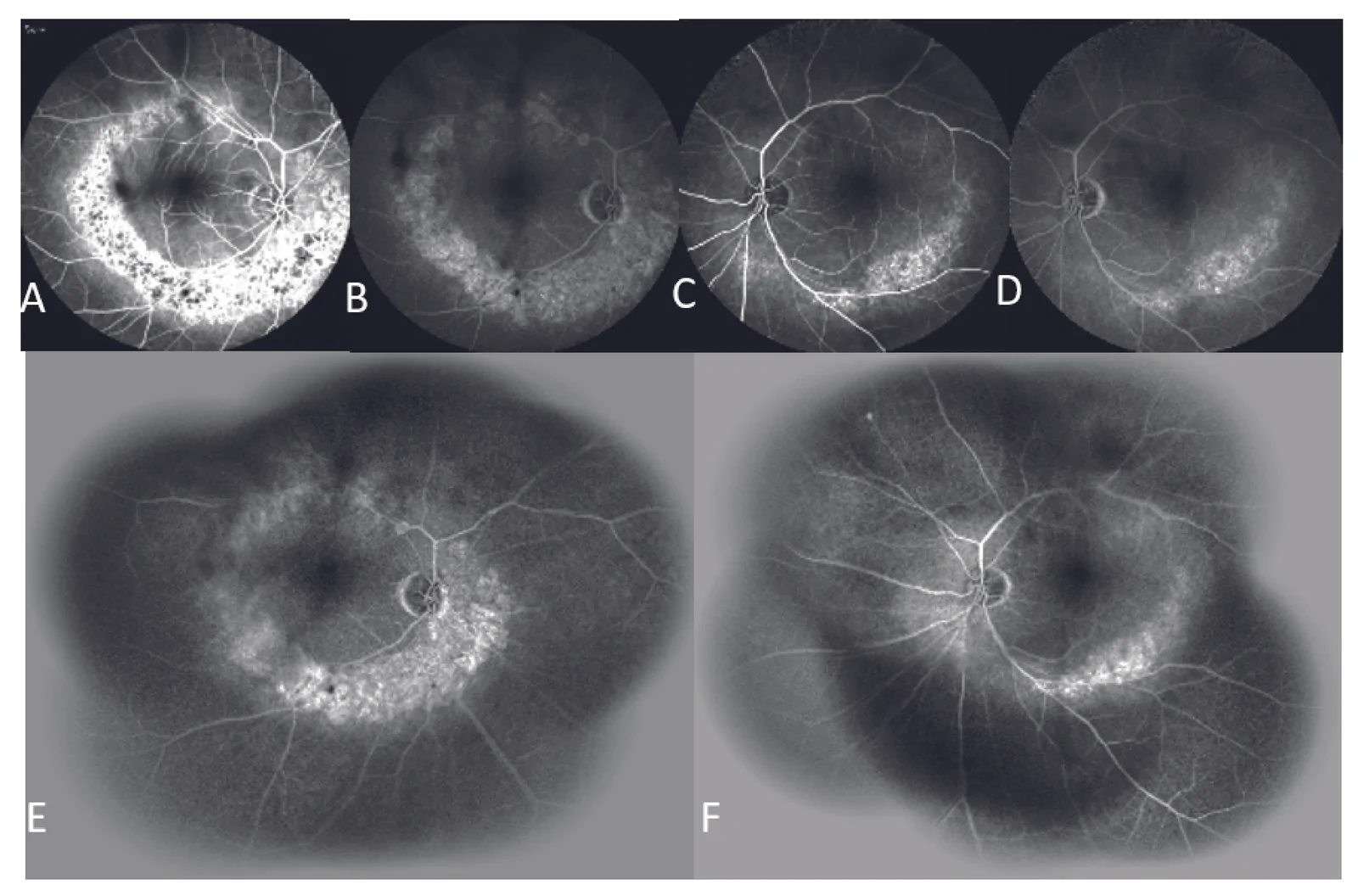
FAG images obtained in the first visit, showing early coarse nodular staining in both eyes along the main superior vascular arcades and the inferior arcade in the right eye – A&C show peak phase with late partially disappearing staining; B&D show late phase; and E&F show wide-field FAG at 7:52 and 5:18 minutes.

**Figure 6:**
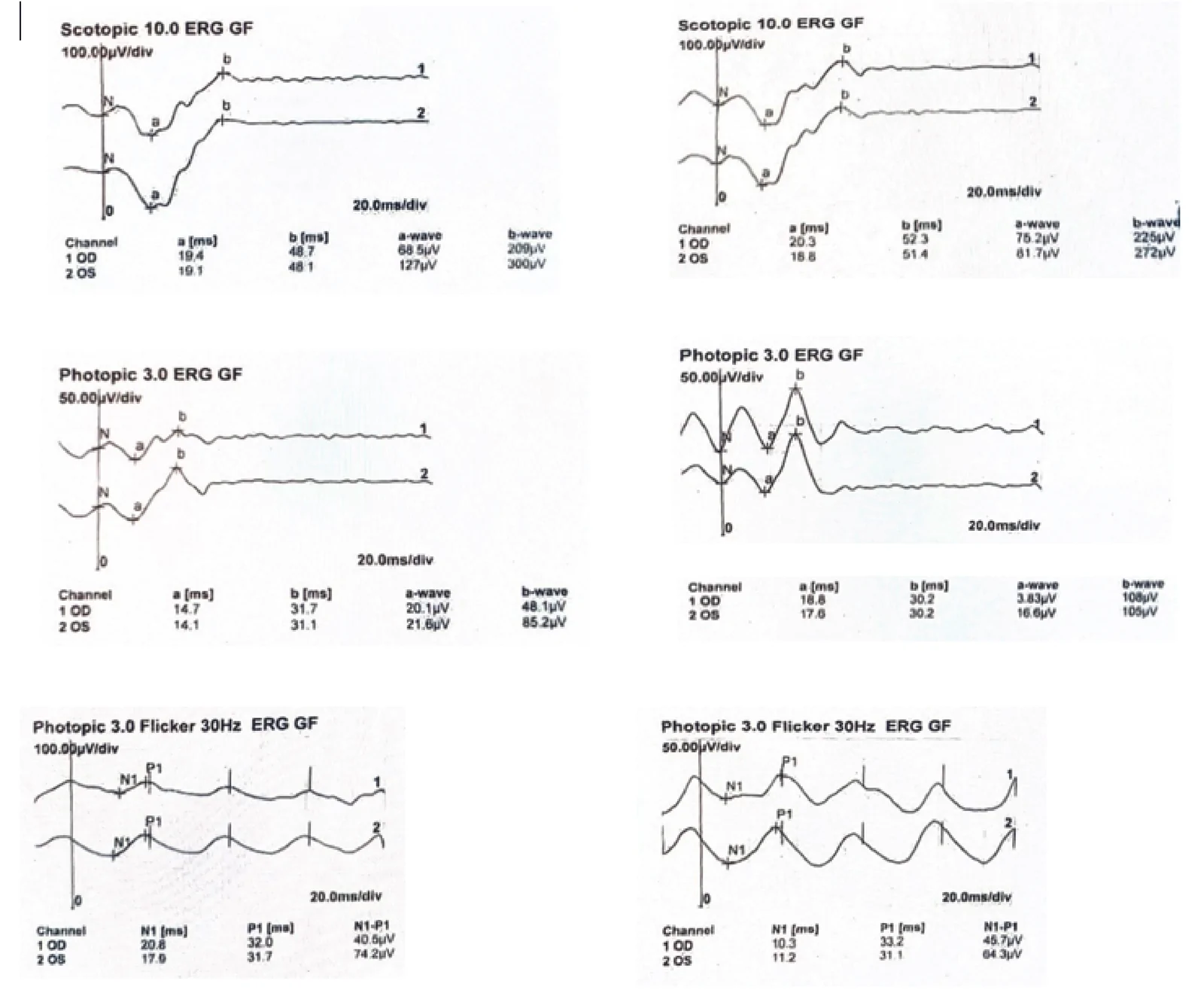
Electroretinogram (ERG): The first column shows initial scotopic and photopic response, both abnormal. The second column shows the scotopic and photopic response 3 months after cessation of ranolazine, both of which had improved.

A 64-year-old male was referred to our clinic with the complaint of a progressive visual field defect in the right eye during the last month. There were no signs of decreased central vision, nyctalopia, or photopsia. He had chronic systemic hypertension and CSAP, was taking aspirin 80 mg and bisoprolol 2.5 mg daily, and had started ranolazine extended-release 500 mg daily, 1 month prior to assessment. He had no history of eye surgery and no family history of ocular pathology. Visual acuity (VA) was 20/20 with - 1.00 D myopic refraction in both eyes and +2.50 D add for near vision. Color vision was normal on Ishihara testing (38 plates edition). Relative afferent pupillary defect (RAPD) was negative. External exam, including ocular motility, was unremarkable. Anterior segment exam was normal, and intraocular pressure was 11 mmHg in the right eye and 13 mmHg in the left eye. Fundus exam showed a normal disc and normal cup-to-disc ratio with peripapillary atrophy and chorioretinal atrophy along the main superior and inferior vascular arcades in the right eye and in the superior temporal arcade in the left eye [Figure 1]. The Humphrey visual field 24-2 test identified a central island preservation and a double arcuate defect scotoma in both eyes, which was specifically more prominent in the inferior hemifield of the right eye [Figure 2]. Swept-source optical coherence tomography (OCT) showed outer nuclear layer (ONL), external limiting (ELM), ellipsoid layer (EL), retinal pigmented epithelium (RPE), and choriocapillaris atrophy in the extrafoveal area, which were more prominent in the right eye [Figure 3]. Fundus auto-fluorescence (FAF) disclosed hypo-autofluorescence along the superior temporal, superior nasal, and distal portions of the inferior temporal vascular arcade in the right eye, in addition to the distal portion of the superior temporal vascular arcade in the left eye [Figure 4]. Fluorescein angiography showed staining in the same area [Figure 5]. Electroretinogram (ERG) revealed decreased photopic and scotopic response [Figure 6].. Since the symptoms coincided with the intake of ranolazine, we suggested that the patient stop the medicine. Three months later, the symptom decreased, and both visual field parameters and ERG findings showed improvement.

##  DISCUSSION

There are multiple known cardiac medications associated with retinal and optic nerve toxicity. Procainamide is known to cause occlusive retinopathy affecting second-order retinal vessels, which can lead to severe vision loss.[5] Amiodarone is repeatedly reported to be associated with bilateral toxic optic neuropathy,[6] and digoxin can cause reversible xanthopsia.[7]

Here, we present a case of potential ranolazine-induced toxicity with significant chorioretinal atrophy along vascular arcades, which was bilateral but asymmetric between and within the two eyes. It showed irreversible structural changes on fundus exam, OCT, and FAF after cessation of the medication but there was partial reversibility in retinal function as demonstrated by improvement in visual field and ERG findings.

There is no other cardiac medication which can cause similar chorioretinal atrophy and significant visual field defect. Procainamide is assumed to cause retinal vascular occlusive disease and amiodarone is reported to lead to toxic optic neuropathy. Our patient did not show any sign of optic neuropathy. VA, color vision, and optic nerve head exam were normal and RAPD was negative. Fundus exam and FAG did not show any sign of vascular accident, but we observed an outer retina, RPE, and choriocapillaris atrophy along vascular arcades.

Several potential mechanisms may explain ranolazine-associated chorioretinal toxicity. RPE cells express potassium (K ${}^{+}$ ) channels, which are crucial to generating membrane potential and regulating cellular volume.[8] Additionally, calcium activated potassium channels (maxi-K) are expressed in both the apical and basolateral membranes of *in vitro* human induced pluripotent stem cell-derived RPE cells. Maxi-K inhibition can reduce apical pigment epithelium-derived factor (PEDF) and basolateral vascular endothelial growth factor (VEGF) secretion.[9] Human donor RPE cells also express the epithelial sodium channel (ENaC), which regulates cellular ion and water homeostasis.[10] The potential cytotoxic effects of the medication and its accumulation in the outer retina and RPE can offer another explanation. This can explain the chorioretinal atrophy along the vascular arcades, which are susceptible to earlier damage due to higher blood-flow perfusion and greater drug accumulation.

Other possible diagnoses for this patient should also be considered, including late-onset retinal dystrophies, autoimmune or paraneoplastic retinopathies, prior inflammatory retinopathy, chronic ischemic retinopathy, and other chronic drug toxicities unknown to us at the time of presentation. However, the temporal association between the visual symptoms and ranolazine initiation, combined with partial improvements following drug cessation, raises the possibility—although it does not establish causality—of ranolazine-induced retinal toxicity.

To our knowledge, this is the first case of presumed chorioretinal toxicity potentially related to ranolazine in the English-language literature. We reported visual field defect concurrent with 1 month of ranolazine intake and partial improvement after cessation of the drug. This was observed over a 3-month follow-up using VF, OCT, FAG, FAF, and ERG.

This report lacks retinal and choroidal histologic findings, including evidence of potential ranolazine accumulation or possible cellular ion channel activity changes. Furthermore, we did not find any documentation of prior ocular examinations or imaging for this patient. Future cellular studies, case series, or large cohort studies can help differentiate our proposed association from an incidental finding.

In summary, we reported a significant visual side effect associated with short-term intake of ranolazine extended-release, which partially improved after cessation but still left permanent sequelae.Further studies can lead to better understanding of the biochemical mechanisms and prevalence of this finding.

##  Ethical Considerations

Written informed consent was obtained from the patient for publication of this case report and accompanying images.

##  Financial Support and Sponsorship

None.

##  Conflicts of Interest

None.

## References

[1] Scirica BM, Morrow DA, Hod H, Murphy SA, Belardinelli L, Hedgepeth CM (2007). Effect of ranolazine, an antianginal agent with novel electrophysiological properties, on the incidence of arrhythmias in patients with non ST-segment elevation acute coronary syndrome: Results from the Metabolic Efficiency With Ranolazine for Less Ischemia in Non ST-Elevation Acute Coronary Syndrome Thrombolysis in Myocardial Infarction 36 (MERLIN-TIMI 36) randomized controlled trial. Circulation.

[2] Reed M, Kerndt CC, Gopal S, Pellegrini MV, Nicolas D (2025). Ranolazine.

[3] Jerling M (2006). Clinical pharmacokinetics of ranolazine. Clin Pharmacokinet.

[4] Koren MJ, Crager MR, Sweeney M (2007). Long-term safety of a novel antianginal agent in patients with severe chronic stable angina: The Ranolazine Open Label Experience (ROLE). J Am Coll Cardiol.

[5] Nichols CJ, Mieler WF (1989). Severe retinal vaso-occlusive disease secondary to procainamide-induced lupus. Ophthalmology.

[6] Wang AG, Cheng HC (2016). Amiodarone-associated optic neuropathy: Clinical review. Neuroophthalmology.

[7] Lawrenson JG, Kelly C, Lawrenson AL, Birch J (2002). Acquired colour vision deficiency in patients receiving digoxin maintenance therapy. Br J Ophthalmol.

[8] Wulff H, Castle NA, Pardo LA (2009). Voltage-gated potassium channels as therapeutic targets. Nat Rev Drug Discov.

[9] Mamaeva D, Jazouli Z, DiFrancesco ML, Erkilic N, Dubois G, Hilaire C (2021). Novel roles for voltage-gated T-type Ca
${}^{2+}$
 and ClC-2 channels in phagocytosis and angiogenic factor balance identified in human iPSC-derived RPE. FASEB J.

[10] Xu P, Zou W, Yin W, Chen G, Gao G, Zhong X (2024). Ion channels research in hPSC-RPE cells: Bridging benchwork to clinical applications. J Transl Med.

